# Viral load responses to HAART is an independent predictor of a new AIDS event in late stage HIV infected patients: prospective cohort study

**DOI:** 10.1186/1479-5876-3-40

**Published:** 2005-10-31

**Authors:** Powel Kazanjian, Wei Wei, Morton Brown, Tejal Gandhi, Kamal Amin

**Affiliations:** 1 Department of Internal Medicine, University of Michigan Health System, Ann Arbor, Michigan, USA; 2 Department of Epidemiology, University of Michigan School of Public Health, Ann Arbor, Michigan, USA

**Keywords:** Viral Load, CD4, HAART, AIDS

## Abstract

**Background:**

A sizeable number of HIV-infected patients receiving HAART do not maintain prolonged virologic suppression. We evaluated long-term HIV viral load (VL) responses to HAART as a risk factor for AIDS events (AE) that is independent of CD4 responses.

**Methods:**

A cohort of patients with pre-therapy CD4 < 200/mm^3 ^who had CD4 and VL measurements for > one year after receiving HAART at a university clinic were prospectively enrolled. Cox proportional multivariate regression model was used to determine whether CD4 and VL responses were independently associated with new AE.

**Results:**

The patient (N = 214) mean baseline CD4 = 92/mm^3^, VL = 219,000 c/mL and follow-up duration 42.3 months (range 13–72 months). A new AE occurred in 56 patients; CD4 cell count response to HAART that remained < 200/mm^3 ^throughout the study period was a significant risk factor for new AE (RR = 9.7–12.5; p < 0.001). Similarly, VL responses that remained > 5,000 c/mL during this period was also a significant risk factor (RR = 6.7–12.8; p = 0.001) that was independent of CD4 response adjusted for <> 200/mm^3^.

**Conclusion:**

Maintaining adequate long-term virologic responses to HAART provides a clinical benefit independent of CD4 responses.

## Background

Despite an overall decline in AIDS-associated illnesses since the introduction of HAART [1,2], some patients receiving combination antiretrovirals remain at risk for developing new AIDS events (AE) [3,4]. Those who have a pre-treatment CD4 cell count < 200/mm^3 ^[5-9] or have insufficient CD4 cell responses to HAART [6] are at risk for progressing to AIDS or dying while receiving therapy. Persistent viremia [10], independent of insufficient CD4 cell count responses [13-16], has also been shown to be a predictor of disease progression. These studies, however, have differed in regards to the VL that they identify as being predictive of disease progression-- > 1,000 c/mL [10], > 7,000 c/mL [13], or > 20,000 c/mL [11]. Furthermore, although these studies have linked short virologic markers in response to HAART with a successful clinical outcome [11-14], the importance of sustaining immunologic and virologic responses to HAART over a more prolonged time period remains to be addressed.

It is important to evaluate whether maintaining adequate responses to long-term HAART treatment has clinical significance for several reasons. First, evaluating whether long-term virologic response is an independent predictor of new AE has relevance for the sizeable number of patients receiving HAART who do not maintain prolonged virologic suppression [1-4,15]. Also, the meaning of persistent viremia is especially pertinent to those who have high-level resistance to antivirals, but nonetheless sustain an immunologic benefit while continuing their failing HIV regimen [16]. Furthermore, evaluating whether immunologic responses to HIV treatment is an independent risk factor for disease progression is applicable to those patients who are unable to mount significant CD4 cell elevations, regardless of whether they have undetectable VL [17]. Thus, our aim was to determine whether long term virologic, as well as immunologic responses to HAART are important and independent predictors of developing a new AE as well as to identify a level of persistent viremia that is predictive of disease progression. We chose patients with significant immune depletion because they are at highest risk for disease progression [3,5].

## Methods

We identified HIV-infected patients treated at the University of Michigan HIV/AIDS Treatment Clinic (UMHATC) who had a baseline CD4 cell count < 200 cells/mm^3 ^before initiating a HAART regimen. The study began on January 31, 1996; patients were enrolled into this prospective cohort from the time therapy was initiated if they received at least twelve months of HIV therapy and were followed to January 1, 2005. The definition of HAART for this study was guided by DHHS guidelines [18]. Patients were included if they were antiviral naïve before starting HAART or if they had previously received one or two nucleoside agents. Patient information was extracted from an electronic database (Solutions™ Patient Management System). The research was carried out according to the principles of the Declaration of Helsinki and the study was approved by the Institutional Review Board of the University of Michigan Health System.

Data on markers of response to HAART (i.e., CD4 and VL levels that were obtained during the course of routine UMHATC practice on an every three month basis), as well as occurrence of a new AE (1993 CDC AIDS Surveillance definition) [19], were extracted from the electronic database system. CD4 cell counts were determined using flow cytometry, and HIV RNA levels were performed using a reverse transcriptase polymerase chain reaction assay (Amplicor; Roche Diagnostics, Branchburg, New Jersey, USA). For purposes of consistency, a VL value of < 400 copies/mL was considered undetectable for the study period, as lower limit changed to < 50 c/mL in 1999 in the middle of our study period. We also recorded assessment of adherence that was entered into our electronic database by nurses, social workers, and physicians, as was our standardized practice during this study period.

To compare baseline characteristics between patients who developed an AE and those who did not, we used histograms to examine distributions of variables that were collected. Appropriate transformations were applied on variables found asymmetric in the following analysis. Baseline quantitative characteristics of patients (e.g. age, CD4, VL) were described using mean and standard deviation, and qualitative characteristics (e.g. gender, presence of AE at baseline) using proportion. A t test was used to compare quantitative characteristics between patients who developed an AE and those who did not. A Chi-square test was used to compare qualitative characteristics between groups.

To determine probabilities of occurrence of a new AE from HAART initiation, we measured time to first AE and used Kaplan-Meier curves. To determine averaged VL trends after initiating HAART we used lowess smooth curves fitted to all patients, as well as to those who developed an AE and those who did not. We then determined VL values for the entire study period, as well as for four specified periods: 1–12 months, 13–24 months, 25–36 months and 37–48 months after initiating HAART.

To determine risk factors for developing a new AE, we used a Cox proportional hazard multivariate regression model. The proportional hazard assumption was then examined by plotting the complementary log-log transformation of the survival functions separately within subgroups. Pre-treatment factors that were examined as predictors for a new AE included demographic factors (e.g. age and gender), prior NRTI use, and presence of AE at baseline. For a given VL measurement at a particular time point after initiation of HAART, persons were considered at risk for newly diagnosed AE for 1–6 months after the time the measurement was made. For each time period, we classified VL responses to HAART (adjusted for a CD4 response of <> 200 cells/mm^3^) into separate categories to determine whether a particular VL category was independently associated with presence of a new AE. The relative risks of new AE for patients in specified VL categories were based on the fitted proportional hazard model and 95% confidence intervals were estimated. All statistical tests were two-sided, and a p-value less than 0.05 was considered statistically significant. Statistical software R 1.7.0 was used for all statistical analysis.

## Results

### Patient Population and AE Description

Two hundred and fourteen patients (21% of 1,014 patients treated at UMHATC during the study period) were included in the analysis. Table 1 gives the demographic and clinical features of the population, including prior nucleoside use and presence of AE at baseline prior to initiating HAART. The mean duration of follow up for the entire population after starting HAART was 42.3 ± 37.4 (range 13–82 months), and a total of 9,288 person-months was included in the analysis. The incidence rate of a first new AE was 6.1/100 person years for the entire population. There was no significant difference in presence of baseline AE (P = 0.14), or prior nucleoside agent use (P = 0.54) between those who developed an AE and those who did not (P = 0.28). Similarly, there was no significant difference in adherence between the two groups; 48 of the 56 patients who developed a new AE were noted to be adherent with their prescribed HAART regimen on a regular basis (86%) as opposed to 143 of the 158 patients who did not (91%). Table 1 shows that patients without an AE had a more brisk mean CD4 increase, more substantial VL decline, and a greater percentage of undetectable VL than those who did develop an AE.

**Table 1:** Demographic, Clinical Features, Mean CD4* and Viral Load** According to Whether an AE Occurred.

Laboratory Value	Patients with AE (n = 56)	Patients without AE (n = 158)
**Demographic**		
Age ± SD (range)	37.9 ± 8.28 (16 – 65.4)	39.4 ± 8.79 (15.2 – 67.8)
HIV risk (no.)	MSM 27	MSM 82
	HTS 7	HTS 34
	IVDU 4	IVDU 12
	Other 22	Other 36
Clinical		
OI (no.) at Baseline (%)	7 (12%)	25 (15%)
Prior NRTI (no.)	12 (21%)	27 (17%)
Baseline Laboratory Values		
CD4	62	107
VL	257,136	196,832
Follow Up Laboratory Values		
*CD4 (Change from Baseline)*	109 *(47)*	369 *(262)*
VL *(Change from Baseline)*	159,517 *(-97,619)*	29,743 *(167, 089)*
Number (%) with Undetectable VL at any point during follow up	46 (82%)	142 (89%)
Number (%) with Undetectable VL	12 (23%)	95 (62%)

*CD4 in cells/mm^3^

**VL in c/mL

Abbreviations: HTS (Heterosexual), NRTI (Nucleoside Reverse Transcriptase Inhibitor).

**Figure 1:**
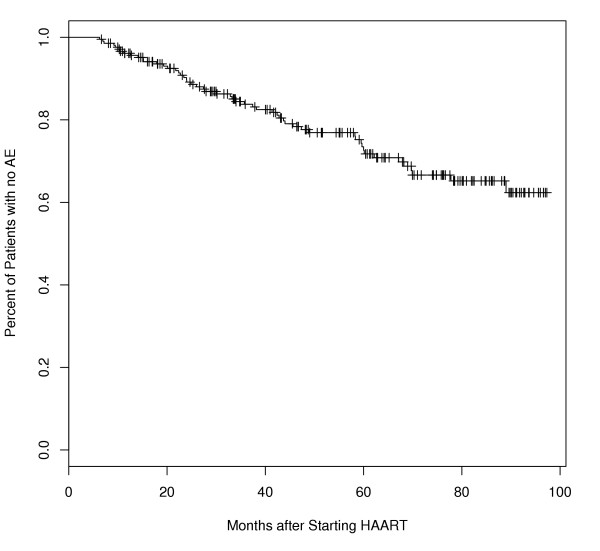
Kaplan-Meier curve of First AE for the entire population. The percentage of patients without an AE is plotted according to time (months) after HAART initiation. The probability of a patient remaining free of a new AE after 48 months of HAART was 78% (95% CI, 71.9, 86).

Fifty-six of the 214 patients (26%) developed a new AE; the mean time from HAART initiation to AE was 26.5 ± 21.2 (range 1 – 84 months). Four patients, all of whom had experienced a new AE, died during the study period. Figure 1 shows that the probability of a patient remaining free of a new AE after 12 and 48 months of HAART was 96.1% (95% CI, 93.5,98.8), and 78% (95% CI, 71.9,86), respectively. There were 60 episodes of new AE that occurred in 56 patients (two patients had 2 new AE and one had 3), as well as AE that were present pre-treatment. Disseminated *Mycobacterium avium *complex (MAC) infection (13 patients), *Pneumocystis jiraveci *pneumonia (PcP, 8), Kaposi's sarcoma (6), candida esophagitis and non Hodgkin's lymphoma (5 each) were the most common AEs. Three of these AEs represented a recurrence of an AE that was present at baseline prior to initiating HAART: PcP (1 patient), MAC (1) and CE (1). 77% of patients in whom MAC was diagnosed were receiving prophylaxis with a macrolide agent, and 88% of those with PcP were receiving pneumocystis prophylaxis. Others included mycobacterial and cryptococcal infections (4 patients each), progressive multifocal leukoencephalopathy and cryptosporidiosis (3 each), HIV encephalopathy and recurrent bacterial infections (2 each), and chronic *Herpes simplex *infections and histoplasmosis (1 each).

### On Treatment CD4 and VL Responses and Association with a New AIDS Event

**Figure 2:**
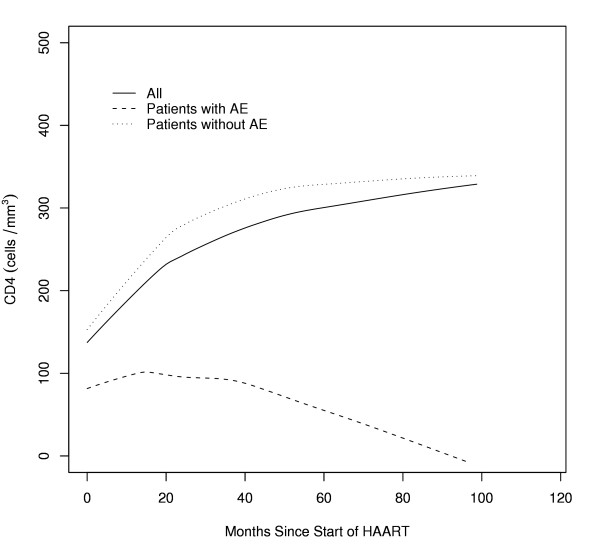
CD4 Response to HIV Therapy for Entire Population and Patients with or without AE. The averaged CD4 responses to HAART throughout the entire study period were determined by lowess smooth curves fitted to all patients for all patients, as well as for those in whom a new AE occurred or did not occur. At each time point during the study, the CD4 response for patients in whom a new AE developed was much lower than for those in whom an AE did not occur.

Figure 2 plots the averaged CD4 responses to HAART throughout the entire study period for all patients, and according to whether a new AE occurred. Figure 2 shows that the CD4 response for patients in whom a new AE developed was much lower throughout the entire time period than for those in whom an AE did not occur. Throughout the study period, the averaged HAART-restored CD4 cell count failed to elevate > 200 cells/mm^3 ^in a significantly higher proportion of patients with a new AE, 85% (48/56 patients), than those without an AE, 8% (13/158 patients) (p < 0.001). Table 2 shows that a CD4 cell count response to HAART that remained < 200/mm^3 ^was a significant risk factor for developing new AE for each 12 month interval after initiating HAART (RR = 9.7–12.5; p < 0.001).

**Table 2:** Risk of Developing New AIDS Event according to CD4 and VL Response Categories.

CD4 Response	**Time After HAART**	**Relative Risk and CI**	**P Value**
Absolute CD4 < 200	(1–12 months)	RR = 4.45 (95% CI: 1.8, 10.9);	p < 0.001
	(13–24 months)	RR = 3.95 (95% CI: 1.62, 9.63);	p = 0.002
	(25–36 months)	RR = 5.19 (95% CI: 1.99, 13.5);	p = 0.001
	(37–48 months)	RR = 3.21 (95% CI: 1.61, 10.8);	p = 0.02
VL Response			
Absolute VL > 1,000	(1–12 mos)	RR = 2.34 (95% CI: 0.87, 6.26);	p = 0.09
	(13–24 mos)	RR = 2.77 (95% CI: 0.81, 9.48);	p = 0.10
	(25–36 mos)	RR = 4.41 (95% CI: 0.99, 19.7);	p = 0.052
	(37–48 mos)	RR = 2.74 (95% CI: 0.71, 9.1);	p = 0.14
Absolute VL > 5,000	(1–12 mos)	RR = 4.66 (95% CI: 2.02, 10.7);	p = 0.001
	(13–24 mos)	RR = 3.24 (95% CI: 1.06, 9.88);	p = 0.03
	(25–36 mos)	RR = 6.41 (95% CI: 1.8, 22.8);	p = 0.004
	(37–48 mos)	RR = 4.23 (95% CI: 1.05, 6.0);	p = 0.05
Absolute VL > 50,000	(1–12 mos)	RR = 3.77 (95% CI: 1.68, 8.43);	p = 0.001
	(13–24 mos)	RR = 2.78 (95% CI: 1.04, 7.39);	p = 0.021
	(25–36 mos)	RR = 2.89 (95% CI: 1.80, 5.51);	p = 0.04

**Figure 3:**
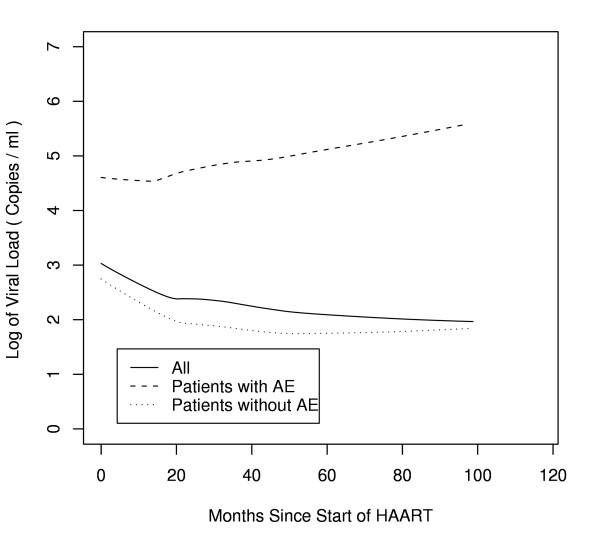
Viral Load Response to HIV Therapy for Entire Population and Patients with or without AE. The averaged VL responses to HAART throughout the entire study period were determined by lowess smooth curves fitted to all patients for all patients, as well as for those in whom a new AE occurred or did not occur. At each time point during the study, the VL reduction for patients in whom a new AE developed was much lower throughout the entire time period than for those in whom an AE did not occur.

Figure 3 plots the averaged VL responses to HAART throughout the entire study period for all patients, and according to whether a new AE developed. Figure 3 shows that the virologic reduction for patients in whom a new AE developed was much lower throughout the entire time period than for those in whom an AE did not occur. Throughout the study period, the averaged HAART-restored VL remained > 5,000 c/mL in a significantly higher proportion of patients with a new AE; 55% (31/56 patients), than those without an AE; 24% (37/158 patients) (p = 0.02). In a multivariate analysis adjusting for CD4 counts of 200 cells/mm^3^, a VL response > 5,000 c/mL was a significant risk factor for developing a first new AE for each 12 month interval after initiating HAART (RR = 6.7–12.8; p = 0.001) (Table 2). Similarly, a VL response that remained > 50,000 c/mL was also a significant risk factor for developing new AE for each 12 month interval after initiating HAART (RR = 3.7–12.6; p = 0.007) (Table 2). However, VL responses that remained > 1,000 c/mL approached but did not reach statistical significance for developing a first new AE (RR 2.34–4.41; p = 0.05–0.14) for each individual 12 month interval after initiating therapy (Table 2).

## Discussion

The present study demonstrates that failure to maintain low levels of viremia is a significant risk factor for HIV disease progression that is independent of CD4 responses. Our results are consistent with studies showing the clinical importance of achieving a VL response at 6–12 months that is independent of CD4 responses [10-14], and stress the benefit of maintaining a durable VL response throughout a 48 month period. The VL level identified as carrying an increased risk for an AIDS event in our study, > 5,000 c/mL, is within the range that some reports identify as independently predictive of disease progression, i.e., > 1,000 c/mL [10], > 7,000 c/mL [13], but lower than another report (20,000 c/mL) [11]. Regardless of the precise value, a reduction in VL that cannot be maintained at low or undetectable levels, a predictor of new AIDS events in our study, is not uncommon for patients receiving HAART [20]. It is unlikely that the difference in VL response between the groups in our study can be attributed to different adherence rates between the two groups. However, because adherence measurement tools were not used as part of routine clinical care, we could not calculate the percentage of each patient's adherence over a long-term basis and then compare this variable between groups.

In fact, several published studies suggest that virologic failure rates are especially high, varying from 20% to 70%, in those who are either treatment-experienced or have low baseline pre-treatment CD4 cell counts and high VL values [21-23]. In one study, for example, lower CD4 counts and higher VL at baseline predicted virologic failure, but there was no clear cut-off value at which the risk started to increase [24]. In another study, baseline CD4 count < 25 cells/mm^3 ^was associated with a significantly higher risk of virologic failure, as was a baseline VL ≥ 100,000 copies/mL [25].

Identifying that long-term virologic response is an independent predictor of new AE has relevance for the sizeable number of patients receiving HAART who cannot maintain prolonged virologic suppression [20]. Also, the meaning of persistent viremia is pertinent to those who have high-level resistance to antivirals, but nonetheless sustain an immunologic benefit while continuing their failing HIV regimen [26]. At present, the outcome and optimal management of these patients remains undefined [27]. Switching to an optimized regimen may not be an available option, since the durability of a salvage regimen selected for patients with high level resistance to many antivirals may be brief. Consequently, some physicians continue a failing regimen despite persistent viremia for patients who maintain an immune benefit until other active treatment options become available. By identifying VL as a risk factor for disease progression that is independent of CD4 response, our study suggests that discordant responders may be at risk for disease progression while being maintained on a virologically failing regimen.

It is not rare for patients, such as those followed in the present study, to first receive HAART after they develop an AIDS event or their CD4 cell count falls below 200/mm^3^. In one report, "late testers" – those whose diagnosis of HIV occurred within 12 months of their AIDS diagnosis, accounted for 39% of persons diagnosed with AIDS in San Francisco between 2001 and 2002 [28]. Also, in a New York study, 27% of persons with newly diagnosed HIV infection were concurrently diagnosed with AIDS [29]. Furthermore, in clinical practice, some patients, despite having been tested earlier, may not have received HAART until a later stage because they may not have wished to take HIV therapy until becoming symptomatic. These reasons, along with sole availability of mono or dual nucleoside agents prior to 1996, accounted for why 21% of patients followed in our practice did not receive HAART until their CD4 count fell below 200/mm^3 ^and therefore met criteria for our study.

Our finding that PCP and MAC remain the most frequent AE in the HAART era is consistent with prior studies [30]. Similarly, our finding that chemoprophylaxis failure among those with the most advanced immunosuppression was the most significant source of new PCP cases is also consistent with a HOPS cohort study [31], along with studies showing that KS and NHL remain the major AIDS-associated malignancies in the HAART era [32-34]. In contrast, our study showed that CMV and cerebral toxoplasmosis occur less often than in the Pre-HAART era. One possible explanation for this observation is that memory CD4 cells, expanded by HAART, may recognize antigens on previously acquired pathogens that persist in a latent state, thereby preventing clinical reactivation. This possibility, if confirmed, suggests that there may be benefits of receiving HAART that are independent of immunologic and virologic responses currently used to monitor therapy.

## Conclusion

In summary, our study emphasizes the clinical benefit of maintaining virologic responses as well as immunologic throughout long-term HAART treatment. Incomplete responses to HAART are not uncommon in clinical practice, and identifying them as risk factors for disease progression emphasizes several treatment options that are available. For example, changing regimens in patients who have persistent virologic replication who have other antiviral options, as recommended by treatment guidelines [35], is supported by identifying VL as an independent predictor of disease progression. Nevertheless, for those with no option other than to remain on their current regimen for prolonged periods of time because of high level drug resistant HIV, clinical progression may eventually occur, even in those who have a CD4 benefit despite persistent viremia. Our findings stress the importance of developing new potent antiretroviral agents in order to sustain the overall decline in AIDS-associated illnesses that has been witnessed since the introduction of HAART.

## Competing interests

The author(s) declare that they have no competing interests.

## Authors' contributions

PK was responsible for study design, data collection, data analysis, and preparation of manuscript.

KA participated in data collection and data analysis.

MB participated in statistical design and analysis

WW participated in statistical design, statistical analysis, and preparation of the manuscript.
